# Assessing myocardial work to predict left ventricular remodeling after ST-segment elevation myocardial infarction: associations with left atrial and left ventricular strain

**DOI:** 10.1186/s44348-026-00081-w

**Published:** 2026-07-29

**Authors:** Gomaa Abdelrazek, Mahmoud H. Mahmoud, Gousay A. Alkhazmari, Tarneem M. Alghamdi, Khaled A. Elkhashab, Hassan M. Ebeid, Moustafa K. Saad

**Affiliations:** 1 Department of Cardiology, Faculty of Medicine, Fayoum University, Fayoum, Egypt; 2 SEHA Sheikh Khalifa Medical City, Abu Dhabi, United Arab Emirates; 3 King Abdulaziz Hospital, Jeddah First Health Cluster, Jeddah, Saudi Arabia; 4 Prince Sultan Cardiac Center Al-Ahsa, Al-Ahsa, Saudi Arabia

**Keywords:** Myocardial work, Left ventricular remodeling, ST-segment elevation myocardial infarction, Left atrial strain, Global longitudinal strain

## Abstract

**Background:**

Left ventricular ejection fraction (LVEF) is the standard marker for systolic function, but it fails to detect subtle changes in myocardial contractility in patients after an ST-elevation myocardial infarction (STEMI). Thus, a new way to evaluate left ventricular (LV) function with high precision is needed to assess disease progression and guide therapeutic interventions. This study evaluates the prognostic significance of left atrial (LA) strain, LV strain, and myocardial work in patients who have undergone primary percutaneous coronary intervention (pPCI) post-STEMI.

**Methods:**

The study population was 100 patients diagnosed with STEMI, all of whom received pPCI for their first STEMI event. Patients were admitted to one of two centers, the cardiology department of Fayoum University in Egypt or the cardiac center at King Fahd University Hospital in Saudi Arabia, between November 2022 and March 2024. Conventional echocardiography and 2D speckle-tracking echocardiography (2D-STE) were performed on each participant within 24 h after pPCI and then repeated at least 6 months later. Myocardial work parameters, the global work index, global constructive work (GCW), global wasted work, and global work efficiency and LA strain parameters were derived from 2D-STE and analyzed for their association with subsequent LV remodeling.

**Results:**

Myocardial work parameters (e.g., global longitudinal strain) were associated with LV remodeling after STEMI treated with pPCI, with GCW showing the most significant association with LV remodeling among the myocardial work indices evaluated. Furthermore, LV remodeling was associated with significantly impaired cardiac structure and function. Patients who developed LV remodeling exhibited higher LV volumes, lower LVEFs, impaired myocardial work parameters, and longer door-to-balloon times than those without remodeling. Additionally, LA function (e.g., LA ejection fraction) was significantly compromised in the remodeling group.

**Conclusions:**

Myocardial work and strain parameters were associated with LV remodeling after STEMI treated with pPCI. GCW demonstrated the strongest association with LV remodeling in the present cohort.

## Background

Survival rates after ST-elevation myocardial infarction (STEMI) have improved substantially during the past few decades; however, both the morbidity and mortality of STEMI remain high [1]. Left ventricular (LV) dysfunction and heart failure are common consequences of STEMI and can cause death [2]. Thus, a way to evaluate LV function with high precision is needed to assess disease progression and guide therapeutic interventions. LV ejection fraction (LVEF) is the standard marker for systolic function, but it fails to detect subtle changes in myocardial contractility [3]. Recent studies have suggested global longitudinal strain (GLS) as a measure of dysfunction, especially when ejection fraction (EF) remains normal [4]. Two-dimensional speckle-tracking echocardiography (2D-STE) can contribute to the quantification of LV global and regional systolic function [5]. Previous studies have shown that GLS can be used to predict LV remodeling and cardiovascular events after STEMI [6, 7]. However, some studies showed that, along with GLS, global circumferential strain and the circumferential strain rate are independent predictors of LV remodeling [8]. Left atrial (LA) volumes and LA function have been recognized as significant predictors of adverse events in a range of cardiovascular diseases [9]. Recently, 2D-STE was shown to be feasible for measuring LA deformations and thus analyzing LA reservoir function (LAr; peak atrial longitudinal strain, PALS) during the LV systolic phase [10]. More recently, LAr measured by PALS has shown good predictive value that is independent of LV GLS and LA volume [11, 12]. However, the additional value of PALS in patients with decreased LV GLS is questionable. A previous study demonstrated that the prognostic value of PALS in patients with acute myocardial infarction (AMI) depends on LV GLS and LA size [13].

## Methods

### Ethics statement

This study was approved by the Ethics Committee of the Faculty of Medicine at Fayoum University. The following provisions ensured the privacy of participants and confidentiality of the data: (1) the patients were given the option of not participating in the study if they did not want to; (2) we used code numbers for each participant, with names and addressed kept in a special file; (3) patients’ names were hidden during the analyses; and (4) the results of the study were used only in a scientific manner.

### Study design and participants

This study evaluates the prognostic significance of LA strain, LV strain, and myocardial work in patients who underwent primary percutaneous coronary intervention (pPCI) after STEMI. It specifically explores LA strain and myocardial work as predictors of LV recovery and long-term cardiovascular health after treatment. By examining the relationship between myocardial strain and work within LA and LV dynamics, this research enhances understanding of cardiac mechanics after myocardial infarction. Ultimately, these insights could inform personalized treatment strategies and improve prognostication for STEMI patients.

The study population was patients who were treated with pPCI for their first STEMI event. Patients were admitted to the cardiology department of Fayoum University in Egypt or the cardiac center at King Fahd University Hospital in Saudi Arabia. The study period spanned from November 2022 to March 2024. Conventional echocardiography and 2D-STE were performed on each participant within 24 h after their pPCI. The key parameters (LV GLS, myocardial work, and PALS) were evaluated to determine their relationship with LV remodeling.

Patients who presented with STEMI, in whom chest pain began within 12 h before the pPCI were included. This presentation aligns with the current clinical guidelines for diagnosing and managing STEMI, which emphasize the importance of intervention within this critical timeframe to optimize outcomes [14]. Patients with the following were excluded: (1) previous myocardial infarction or coronary artery bypass; (2) significant valvular dysfunction; (3) ventricular arrhythmia; (4) atrial fibrillation or paced rhythm; (5) noncardiac disease with a life expectancy of < 1 year; or (6) poor echocardiography windows or bad image quality.

### Patient workups

A thorough medical and surgical history of each patient was obtained to establish baseline health information and previous interventions. Within the first hour after admission, the following clinical data were recorded: age, sex, symptom onset, and cardiovascular risk factors (smoking status, hypertension, and diabetes mellitus). Heart rate, blood pressure, body mass index (BMI), and body surface area were measured to assess the overall health status and risk profile of each patient. Routine laboratory investigations assessed hemoglobin A1c (HbA1c), lipid profile, and serum creatinine levels to evaluate metabolic and renal function. All patients received treatment in accordance with current cardiology guidelines. Before the pPCI, patients were given a loading dose of acetylsalicylic acid (ASA), 180 mg of ticagrelor, and 100 IU/kg of heparin (with a maximum dosage of 5,000 IU) to ensure optimal antiplatelet and anticoagulant therapy (American College of Cardiology, 2021, Guidelines for the Management of ST-Elevation Myocardial Infarction).

### Echocardiography and strain analyses

Echocardiographic data were obtained using a GE Vivid E95 ultrasound machine (GE Healthcare). Echocardiographic images were obtained by recording three consecutive heart cycles during apnea, in accordance with the guidelines of the American Society of Echocardiography [5]. LV end-systolic volume (LVESV), LV end-diastolic volume (LVEDV) and LVEF were determined using the biplane Simpson method in four-, and two-chamber views.

The LV was divided into 16 segments based on the American Society of Echocardiography model. Each segment corresponds to a specific region of the LV, allowing for detailed assessment of regional wall motion. Echocardiographic images were reviewed, and the motion of each LV segment was assessed throughout the cardiac cycle (systole and diastole). The wall motion of each segment was graded according to subjective assessments of wall motion amplitude and changes in LV thickness at systole [15]:

- Score 1 (normal or hyperkinetic motion): Normal contraction with inward motion of the myocardium during systole.
- Score 2 (hypokinetic motion): Reduced contraction or wall thickening, compared with adjacent segments.
- Score 3 (akinetic motion): Absence of contraction with no wall motion.
- Score 4 (dyskinetic motion): Paradoxical outward motion during systole, indicating abnormal bulging or aneurysmal formation.

The wall motion score index is defined as the sum of the segment score ratings divided by the number of segments scored. It is used in echocardiography to assess and quantify regional wall motion abnormalities in the LV and to evaluate myocardial function and identify areas of impaired contraction or hypokinesia. It is obtained from echocardiographic images of the LV taken from standard imaging views, particularly the parasternal long-axis, parasternal short-axis, and apical views [16, 17].

Pulsed-wave Doppler variables were measured at the tip of the mitral valve leaflets in an apical four-chamber view during diastole. The peak velocity of early (E) and late (A) diastole and the mitral valve deceleration time were measured, and the E/A ratio was calculated. The myocardial peak early velocity (e’) was measured at the lateral and medial mitral annulus. For the LV strain analysis, 2D echocardiographic images were obtained from four-, three-, and two-chamber and mid-ventricular short-axis views with frame rates of 60 to 90 frames per second. The LV endocardial and epicardial borders were initially traced at end-diastole, and the software automatically tracked the region of interest on the myocardium. The longitudinal peak systolic strain (LPSS) was obtained for all 16 segments, and the GLS was calculated as the average of the observed segmental values of LPSS from the apical four-, three-, and two-chamber views.

For the LA function analysis, the biplane Simpson method was used. The LA maximum volume at LV end-systole (LAV_max_), LA minimum volume at LV end-diastole (LAV_min_), and LA volume before the active atrial contraction at the onset of the P wave (LAV_preA_) were obtained from apical four- and two-chamber views. All LA volumes were indexed to the body surface area [18]. From those volumes, the indices of LA mechanical function were calculated as follow.

1. Total atrial emptying fraction: LA total EF = ((LAV_max_ – LAV_min_)/LAV_max_) × 100
2. Active atrial emptying fraction (an index of LA active contraction): LA active EF = ((LAV_preA_ – LAV_min_)/LAV_preA_) × 100
3. Passive atrial emptying fraction (an index of LA conduit function): LA passive EF = (LAV_max_ – LAV_preA_)/LAV_max_) × 100.
4. Atrial expansion index of reservoir function [19]: LA expansion index = (LAV_max_ – LAV_min_)/LAV_min_ × 100

For the 2D-STE analysis of LA function, 2D grayscale images were obtained in apical four- and two-chamber views, consistent with the software used to analyze LV strain. Quantification of myocardial work was performed using a commercially available software package (Echopac ver. 202, GE Healthcare). As proposed by Russel et al. [20], myocardial work was estimated as the area of the pressure-strain loops, which were derived by a combination of STE-derived LV strain data and noninvasive LV pressure curves. Peak systolic LV pressure was assumed to be equal to the brachial systolic blood pressure (SBP) measured with a cuff manometer.

To measure PALS (LAr), the beginning of the QRS wave on the electrocardiogram was used as a reference point [10]. After selecting the cardiac cycle, the LA endocardial border was manually traced to automatically create a region of interest covering the thickness of the LA myocardium at 12 atrial segments. PALS values were estimated in each LA segment from two apical views, and the mean was used as the global PALS value.

### Myocardial work in echocardiography

Myocardial work is an emerging tool in echocardiography that incorporates LV afterload into a GLS analysis. Myocardial work correlates with myocardial oxygen consumption. In this study, work efficiency was also assessed. Myocardial work has been evaluated in patients with a variety of clinical conditions to assess what value it adds to LVEF and GLS data.

#### Remodeling methods

Myocardial remodeling describes structural and functional changes in the heart, often occurring in response to stimuli such as pressure or volume overload, ischemia, or pathological conditions.

#### Non-remodeling methods

These methods assess myocardial function and work irrespective of significant structural changes in the heart. For example, LVEF, stroke volume, and contractility parameters can be used to evaluate myocardial function without specific reference to remodeling.

### Principles of myocardial work

The pressure–volume loop is created from measurements in the cardiac catheterization laboratory, and it is a rectangular representation of the phases of the cardiac cycle: isovolumic contraction, systolic ejection, isovolumic relaxation, and diastolic filling. The ratio of the mechanical energy that the myocardium imparts to the outgoing blood to the total energy consumption depends on loading conditions [21]. The slope from the start of the curve to the peak pressure at the end of LV ejection represents the contractility of the heart. The area of the LV pressure–volume loop reflects stroke work and myocardial oxygen consumption. Myocardial contraction has three components. The basal and apical segments contract in opposite directions, with the subepicardium contracting in a right-handed helix and the subendocardium contracting in a left-handed helix [22]; those contractions create circumferential strain. The myocardial fibers shorten from the apex to base in systole; GLS tracks this longitudinal shortening. As the myocardium shortens, it also thickens, which creates positive radial strain; this thickening of the myocardium in systole can readily be seen on transthoracic parasternal short-axis echocardiographic views of the LV. A vendor-specific method for assessing myocardial work was developed to include afterload in the GLS analysis. Given the challenge of calculating myocardial force, pressure is used as a surrogate, and the area of the LV pressure–volume loop is used as an index of myocardial work [20].

Peak LV pressure was estimated using noninvasive cuff blood pressure. This method was evaluated by Russell et al. [20] in an experimental animal study. The noninvasively estimated LV pressure curve was first validated in a dog model using invasive hemodynamic monitoring in different clinical conditions (left bundle branch block and AMI). Peak LV pressure was estimated using brachial artery cuff pressure in this study. The LV pressure reference curve was calculated by pooling single-cycle pressure traces using the following three steps:

1. Timing mitral and aortic valve opening and closing by echocardiography.
2. Stretching or compressing raw data traces to correlate with the valvular event times.
3. Stretching the traces vertically to the appropriate peak value.

### Acquisition steps

Myocardial work assessment was performed with a vendor-specific algorithm. The initial step for acquiring the myocardial work value is obtaining transthoracic views for a GLS analysis. The three standard apical views were acquired at a frame rate of > 40 frames per second with image quality adequate to visualize the myocardial borders. Valvular event times were assessed automatically by machine or adjusted manually through a visual assessment of the apical long-axis view. Automated functional imaging was used to calculate GLS in the apical two-, three-, and four-chamber long-axis views. Those outlines were adjusted manually to conform to the myocardium. That analysis generated a bull’s eye GLS plot, and GLS values were then calculated.

The next step in acquiring the myocardial work value is the noninvasive measurement of SBP from the arm using a sphygmomanometer. Peak LV systolic pressure is estimated to be the systolic cuff pressure, which should be measured at the time of image acquisition. This pressure is then input into the echocardiography machine for the myocardial work analysis. Myocardial work augments automated functional imaging with dynamic LV pressures. Once the blood pressure is put into the software, a myocardial work bull’s-eye plot is created, similar to GLS. Four values are calculated: global work index (GWI), global constructive work (GCW), global wasted work (GWW), and global work efficiency (GWE), which are defined as follows [23]:

- GWI: Average myocardial work based on the pressure-strain loop.
- GCW: Positive work performed by a segment in systole and negative work (segment lengthening) during isovolumic relaxation.
- GWW: Negative work (segment lengthening) during systole and positive work (segment shortening) during isovolumic relaxation.
- GWE: GCW/(GCW + GWW). As with GLS, color-coding is used to visualize the differences among high, normal, and reduced myocardial work.

Each segment was analyzed individually to produce the myocardial work indices. The pressure-strain loop was created globally and for each individual segment.

### Follow-up

At least 6 months after STEMI, each patient was subjected to conventional echocardiography. LV remodeling, assessed by echocardiography, was defined as an LVEDV increase of > 20% compared with the baseline echocardiographic data [24]. Major adverse clinical events were a composite of death from any cause, hospitalization for heart failure, and reinfarction, and their occurrence was determined by both clinical visits and telephone calls hospitalization for heart failure followed the exacerbation of exertional dyspnea; the most typical symptom is pulmonary congestion, and it is treated with intravenous diuretics. Reinfarction was defined as chest pain, elevated cardiac enzyme levels, and obvious changes on an electrocardiogram [25].

### Statistical analysis

The statistical analyses were conducted using IBM SPSS ver. 26 (IBM Corp). The quantitative variables (i.e., demographics and clinical and echocardiographic measurements) are presented as mean ± standard deviation and were compared between groups using unpaired Student t-test. Qualitative variables are presented as frequencies and percentages, and they were analyzed using chi square or Fisher exact tests, as appropriate. A two-tailed *P*-value of <0.05 was considered statistically significant. The echocardiographic myocardial work parameters were analyzed using receiver operating characteristic curve graphs to examine their role in predicting LV remodeling.

## Results

Our study population comprised 100 patients diagnosed with STEMI and treated with pPCI who had follow-up echocardiography data from at least 6 months after their STEMI event (average of 11.3 ± 5.0 months). Patients were divided into two groups, the LV remodeling group and the non-remodeling group. LV remodeling, as evaluated by echocardiography, was defined as an increase in LVEDV of more than 20%, compared with the baseline measurement [24]. The clinical characteristics of the two groups were analyzed separately to identify significant differences and associations related to cardiac outcomes. This classification is consistent with current cardiac guidelines that emphasize the importance of LVEDV as a predictor of post– myocardial infarction remodeling and overall prognosis [25].

The mean age of the included patients was 55.16 ± 13.2 years, and most of them (92%) were male, with a mean BMI of 27.8 kg/m^2^ (overweight). Some patients were diabetic (47%), hypertensive (47%), smokers (38%), or dyslipidemic (35%), and 18% were diabetic and hypertensive, 16% were hypertensive and dyslipidemic, 14% were diabetic and dyslipidemic, and 8% had all three comorbidities. When comorbidity status (e.g., hypertension) was assessed with smoking status, 16% were found to be smokers and dyslipidemic, 14% were smokers and hypertensive, and 8% were smokers and diabetic. Clinically, the patients’ presenting mean heart rate was 81.3 ± 19.4/min, SBP was 140.0 ± 21.6 mmHg, and diastolic blood pressure (DBP) was 90 ± 18 mmHg. Age, sex, weight, height, and BMI did not differ significantly between the groups, indicating that the groups were suitable for comparison (Table 1). Similarly, comorbidities, SBP, DBP, and heart rate differed insignificantly between the groups.

**Table 1:** Demographic data and clinical characteristics (*n*=100)

Characteristic	Remodeling group (*n* = 48)	Non-remodeling group (*n* = 52)	*P*-value
Age (yr)	55.71 ± 11.48 (29–79)	54.62 ± 15.03 (30–82)	0.686
Sex			0.906
Male	44 (91.7)	48 (92.3)	
Female	4 (8.3)	4 (7.7)	
Weight (kg)	75.32 ± 12.9 (60–103)	74.62 ± 10.97 (60–91)	0.770
Height (m)	1.64 ± 0.08 (1.48–1.86)	1.64 ± 0.06 (1.55–1.75)	0.739
Body mass index (kg/m^2^)	28.29 ± 4.15 (21.26–35.79)	27.42 ± 4.08 (21.22–34.96)	0.294
Diabetes	23 (47.9)	24 (46.2)	0.860
Hypertension	19 (39.6)	28 (53.8)	0.153
Dyslipidemia	17 (35.4)	18 (34.6)	0.933
Smoking	18 (37.5)	20 (38.5)	0.921
Systolic blood pressure (mmHg)	142.15 ± 19.93 (114–189)	137.9 ± 23.28 (95–181)	0.332
Diastolic blood pressure (mmHg)	91.52 ± 15.38 (67–127)	88.29 ± 20.47 (55–129)	0.377
Heart rate (/min)	78.92 ± 20.77 (54–140)	83.73 ± 18.1 (49–120)	0.219

Values are presented as mean ± standard deviation (range) or number (%)

The presence of anterior wall myocardial infarction and multivessel disease were significantly higher in the remodeling group than the non-remodeling group (*P* = 0.045 and *P*=0.009, respectively). Furthermore, compared with the remodeling group, the non-remodeling group had a 6.62 ± 2.18-min decrease in the mean door-to-balloon time and a 0.16 ± 0.08 decrease in mean creatine, and both decreases were significant (*P* < 0.001 and *P*=0.001, respectively). On the other hand, the glomerular filtration rate, HbA1c, and creatinine kinase-MB values differed insignificantly between the groups (Table 2).

**Table 2:** Laboratory and diagnostic findings (*n*=100)

Variable	Remodeling group (*n* = 48)	Non-remodeling group (*n* = 52)	*P*-value
Door-to-balloon time (min)	51.21 ± 8.28 (39–74)	57.83 ± 10.46 (29–73)	< 0.001^*^
Glomerular filtration rate (mL/min/1.73^2^)	99.23 ± 12.86 (64.06–126.15)	99.06 ± 19.87 (80.27–179.78)	0.961
Creatine (mg/dL)	0.95 ± 0.19 (0.56–1.43)	1.11 ± 0.27 (0.46–1.61)	0.001^*^
HbA1c (%)	7.34 ± 2.1 (5.3–13.5)	7.1 ± 2.62 (0–12.9)	0.620
CK-MB (μ/L)	976.85 ± 1,347.39 (109–6,646)	901.95 ± 809.12 (109.5–3,307)	0.735
Anterior wall MI	29 (60.4)	21 (40.4)	0.045^*^
Multivessel disease	22 (45.8)	11 (21.2)	0.009^*^

Values are presented as mean ± standard deviation (range) or number (%)

*HbA1c* Hemoglobin A1c, *CK-MB* creatinine kinase-MB, *MI* myocardial infarction

^*^P < 0.05

**Fig. 1:**
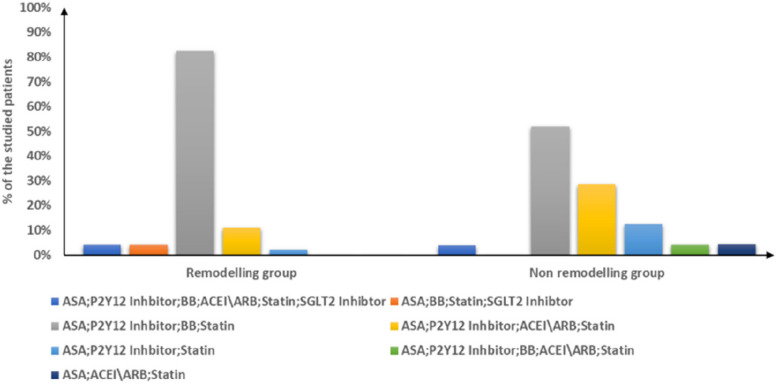
Medication during initial hospitalization. ACEI, angiotensin-converting enzyme inhibitor; ARB, angiotensin receptor blocker; ASA, acetylsalicylic acid; BB, β-blocker; SGLT2, sodium-glucose cotransporter 2

A regimen of ASA, a P2Y12 inhibitor, β-blocker, and a statin was used in 38 patients (82.61%) in the remodeling group and 26 (52%) in the non-remodeling group. The non-remodeling group had 14 patients (28.57%) treated with a regimen of ASA, a P2Y12 inhibitor, angiotensin-converting enzyme inhibitor/angiotensin receptor blocker, and a statin, which was triple the number of patients treated with that regimen in the remodeling group. Therefore, the difference in pharmaceutical regimens was statistically significant (*P* = 0.014) (Fig. 1).

The initial LVESV and LVEDV were significantly higher in the remodeling group than in the non-remodeling group (*P* = 0.002 and *P* = 0.006, respectively), but the initial LVEF did not significantly differ between the groups. The follow-up LVESV and LVEDV remained significantly higher in the remodeling group (both *P* < 0.001), and LVEF became significantly lower (*P* = 0.008). The percentage change in LVESV differed insignificantly between the groups, but that in LVEDV was significantly higher in the remodeling group (*P* < 0.001), and that in LVEF was significantly lower (*P* = 0.047). In addition, the myocardial work parameters (i.e., GLS, GWI, GCW, GWW, and GWE) differed significantly between the groups (all *P* < 0.05). The LA parameters (initial LAr, LA conduit function [LAcd], LA contraction [LAct], and deceleration time) differed significantly between groups (all *P* < 0.05). Furthermore, the initial LA volume maximum biplane views (LAV_maxBiP_) was significantly higher in the remodeling group (*P* = 0.003), and LAEF was significantly lower (*P* = 0.002). The follow-up LAr, LAcd, and LAct also differed significantly between groups (all *P* < 0.05), and LAV_maxBiP_ was significantly higher (*P* < 0.001) and LAEF was significantly lower (*P* = 0.019) in the remodeling group. The percentage change in LAr, LAcd, and LAct differed significantly between the groups (all *P* < 0.05), and LAV_maxBiP_ was significantly higher (*P* = 0.003) and LAEF significantly lower (*P* = 0.002) in the remodeling group. The E/A ratio differed insignificantly between groups; however, the E/e’ ratio was significantly higher in the remodeling group (*P* = 0.021) (Table 3).

**Table 3:** Echocardiographic findings (*n*=100)

Finding	Remodeling group (*n* = 48)	Non-remodeling group (*n* = 52)	*P*-value
LVESV (mL)			
Initial	63.32 ± 40.22 (21.5 to 168)	43.45 ± 17.46 (19.1 to 102)	0.002^*^
Follow-up	68.85 ± 31.77 (26.8 to 141)	41.76 ± 14.73 (17 to 87)	< 0.001^*^
% Change	5.53 ± 38.1 (–121.6 to 60.9)	1.15 ± 6.95 (–18.4 to 15.3)	0.417
LVEDV (mL)			
Initial	99.66 ± 44.73 (46.8 to 258.5)	80.08 ± 22.79 (43.8 to 156)	0.006^*^
Follow-up	131.60 ± 34.44 (65.6 to 198.1)	81.23 ± 24.03 (43.8 to 162)	< 0.001^*^
% Change	31.94 ± 27.88 (–60.4 to 83.1)	–1.69 ± 8.52 (–23 to 13)	< 0.001^*^
LVEF (%)			
Initial	46.00 ± 11.91 (26 to 67)	49.23 ± 10.06 (32 to 66)	0.145
Follow-up	44.90 ± 12.62 (24 to 63)	51.10 ± 10.22 (33 to 68)	0.008^*^
% Change	–1.10 ± 7.89 (–11 to 30)	1.87 ± 6.87 (–8 to 21)	0.047^*^
GLS (mmHg%)			
Initial	–10.70 ± 3.21 (–10)	–13.40 ± 3.65 (–13)	0.004^*^
Follow-up	–12.00 ± 3.33 (–13)	–14.60 ± 3.90 (–13)	0.002^*^
% Change	–1.20 ± 3.33 (–10 to 3)	–2.40 ± 2.96 (–5 to 2)	0.006^*^
GWI (mmHg%)			
Initial	1,086.69 ± 373.60 (465 to 1,309)	1,245.00 ± 406.60 (431 to 1,725)	0.046^*^
Follow-up	1,071.80 ± 398.50 (168 to 1,129)	1,328.00 ± 473.00 (431 to 1,657)	0.023^*^
% Change	–14.85 ± 567.75 (–1,670 to 1,147)	170.94 ± 333.57 (–326 to 916)	0.047^*^
GCW (mmHg%)			
Initial	1,336.69 ± 510.24 (1,367 to 664)	1,465.00 ± 405.70 (536 to 2,008)	0.038^*^
Follow-up	1,355.48 ± 466.00 (760 to 1,589)	1,534.66 ± 556.00 (535 to 1,829)	0.045^*^
% Change	381.64 ± 128.96 (–510 to 1,182)	347.71 ± 165.77 (–216 to 1,223)	0.044^*^
GWW (mmHg%)			
Initial	224.96 ± 82.66 (77 to 365)	240.52 ± 142.81 (66 to 653)	0.002^*^
Follow-up	228.08 ± 128.33 (46 to 537)	241.00 ± 117.00 (59 to 450)	0.004^*^
% Change	123.09 ± 3.13 (–243 to 389)	146.83 ± 30.73 (–368 to 296)	0.026^*^
GWE (%)			
Initial	78.88 ± 8.56 (60 to 82)	89.60 ± 7.85 (63 to 94)	0.003^*^
Follow-up	84.73 ± 8.96 (68 to 97)	92.38 ± 7.18 (73 to 96)	0.039^*^
% Change	8.82 ± 1.67 (–12 to 21)	7.56 ± 2.73 (–7 to 18)	0.049^*^
LAr			
Initial	14.77 ± 5.23 (11 to 22)	20.65 ± 5.64 (9 to 30)	0.002^*^
Follow-up	19.81 ± 7.80 (7 to 37)	26.80 ± 5.49 (9 to 33)	0.004^*^
% Change	19.52 ± 5.23 (11 to 28)	24.98 ± 5.61 (9 to 30)	0.003^*^
LAcd			
Initial	–12.65 ± 4.69 (–20 to –4)	–15.77 ± 4.32 (–23 to –8)	0.001^*^
Follow-up	–10.02 ± 4.99 (–19 to –1)	–13.9 ± 6.01 (–34 to –1)	0.003^*^
% Change	–14.65 ± 4.63 (–22 to –4)	–16.54 ± 4.32 (–25 to –8)	0.004^*^
LAct			
Initial	–7.15 ± 3.25 (–13 to –2)	–10.4 ± 3.75 (–16 to –1)	0.044^*^
Follow-up	–9.38 ± 4.48 (–18 to –2)	–11.9 ± 4.17 (–20 to –3)	0.034^*^
% Change	–9.15 ± 3.20 (–15 to –2)	–12.3 ± 3.71 (–18 to –1)	0.036^*^
LAV_maxBiP_			
Initial	63.69 ± 17.16 (31 to 92)	51.56 ± 22.49 (27 to 105)	0.003^*^
Follow-up	74.17 ± 20.57 (20 to 107)	49.00 ± 21.53 (23 to 119)	< 0.001^*^
% Change	63.69 ± 17.16 (31 to 92)	51.56 ± 22.49 (27 to 105)	0.003^*^
LAEF (%)			
Initial	41.27 ± 11.21 (9 to 63)	47.71 ± 8.70 (22 to 59)	0.002^*^
Follow-up	39.17 ± 10.22 (14 to 52)	43.94 ± 9.84 (25 to 65)	0.019^*^
% Change	41.27 ± 11.21 (9 to 63)	47.71 ± 8.7 (22 to 59)	0.002^*^
Initial deceleration time (msec)	175.35 ± 53.32 (86 to 299)	187.75 ± 51.77 (101 to 340)	0.241
E/A ratio	1.01 ± 0.31 (0.53 to 1.99)	1.11 ± 0.27 (0.53 to 1.7)	0.098
E/e’ ratio	10.13 ± 2.92 (7 to 19.8)	8.88 ± 2.38 (5.6 to 13)	0.021^*^

Values are presented as mean ± standard deviation (range)

*GCW* global construction work, *GLS* global longitudinal strain, *GWE* global work efficiency, *GWI* global work index, *GWW* global wasted work, *LAcd* left atrial conduit function, *LAct* left atrial contraction, *LAEF* left atrial ejection fraction, *LAr* left atrial reservoir function, *LAV*_*maxBiP*_ left atrial volume maximum biplane views, *LVEDV* left ventricular end‐diastolic volume, *LVEF* left ventricular ejection fraction, *LVESV* left ventricular end-systolic volume

^*^P<0.05

**Fig. 2:**
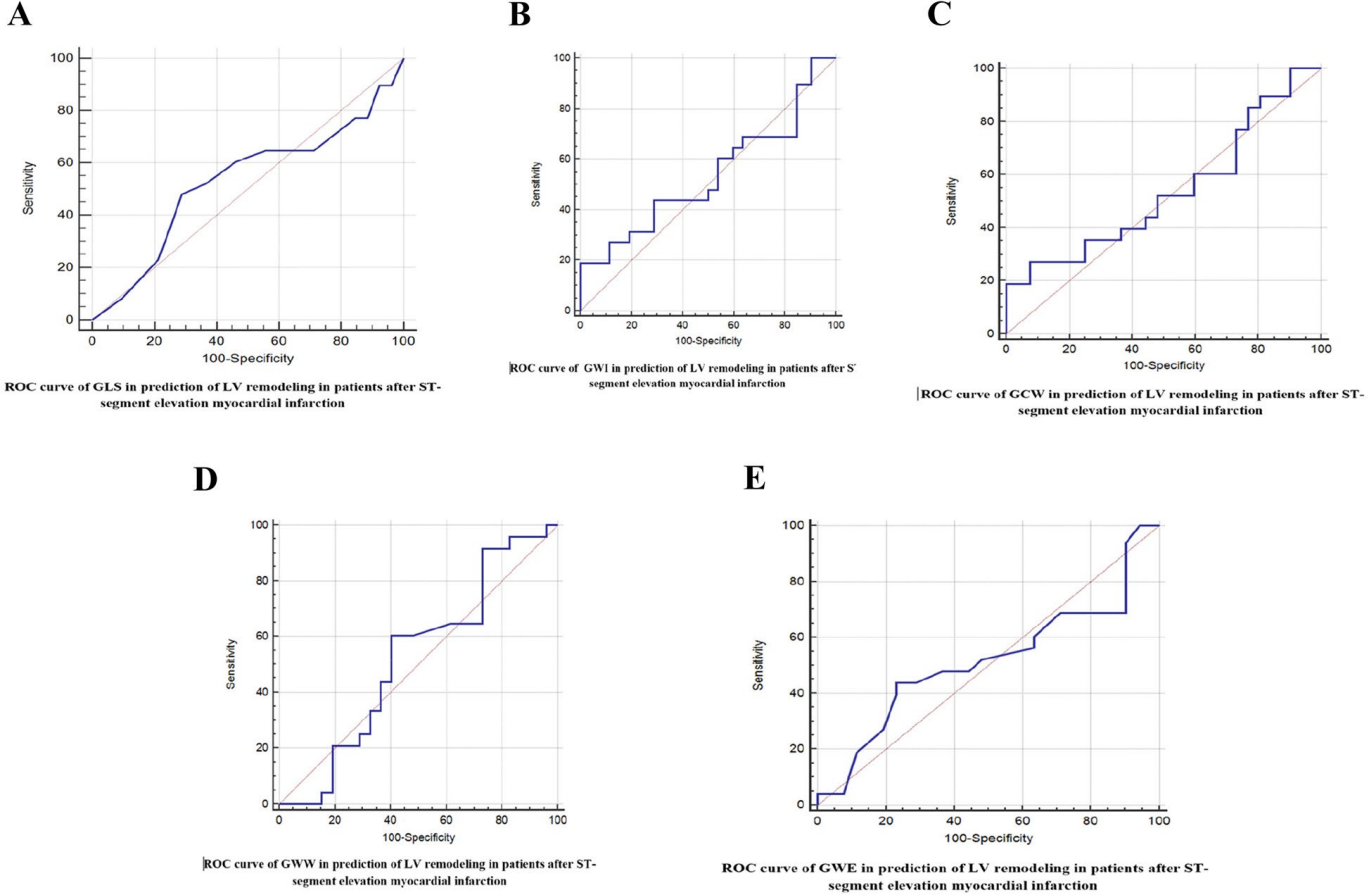
Predictors of left ventricular (LV) remodeling demonstrated in receiver operating characteristic (ROC) curves. Role of (**A**) global longitudinal strain (GLS), **B** global work index (GWI), **C** global constructive work (GCW), **D** global wasted work (GWW), and **E** global work efficiency (GWE)

Receiver operating characteristic (ROC) curves are depicted in Fig. 2 and Table 4 shows that the high sensitivity and positive predictive value (PPV) of GLS and GWI make them valuable tools for identifying patients at risk for LV remodeling after STEMI. Early identification of high-risk patients can help guide therapeutic decisions and monitoring. However, the moderate specificity and low negative predictive value (NPV) indicate that although those variables can effectively identify patients at risk, they are not adequately reliable in identifying patients who will not experience remodeling. Therefore, using them alone could lead to unnecessary anxiety or interventions for some patients. The area under the curve (AUC) suggests that although they show promise, they should not be used as sole determinants for predicting LV remodeling. Additional assessments or combined metrics could enhance their predictive accuracy.

**Table 4:** Myocardial work parameters

Variable	Cutoff	Sensitivity (%)	Specificity (%)	PPV (%)	NPV (%)	AUC	*P*-value
GLS (mmHg%)	–15	84.58	64.23	81.7	57.5	0.529	0.047
GWI (mmHg%)	758	81.58	66.38	67.0	55.3	0.536	0.049
GCW (mmHg%)	1,029	88.42	77.38	75.3	52.5	0.536	0.045
GWW (mmHg%)	180	64.58	38.46	49.2	54.1	0.522	0.004
GWE (%)	79	60.42	36.54	46.8	50.0	0.515	0.036

*AUC* area under the curve, *GCW* global construction work, *GLS* global longitudinal strain, *GWE* global work efficiency, *GWI* global work index, *GWW* global wasted work, *NPV* negative predictive value, *PPV* positive predictive value

On the other hand, the relatively high sensitivity and specificity of GCW suggest a potential association with LV remodeling after STEMI. Therefore, combining GCW with other clinical, echocardiographic, and biochemical parameters could improve the overall assessment of patients following STEMI. GWW and GWE also demonstrated statistically significant associations with LV remodeling, so they could be interpreted in conjunction with other established clinical and imaging markers, rather than as isolated measures.

## Discussion

It is well known that STEMI outcomes have improved dramatically in recent years because of the introduction of modern thrombolytic drugs and PCI. However, LV remodeling still occurs in 30% to 35% of patients [26]. Progressive changes in myocardial wall and ventricular structure, including expansion of the infarct region, wall thinning, and ventricular dilation in the noninfarcted region, can be followed by adverse cardiovascular events and increase the mortality rate [27]. The 2D-STE can quantify regional LV function from standard grayscale 2D echocardiographic images by estimating myocardial strain independent of the angle of incidence [28]. It has recently been validated against sonomicrometry and tagged magnetic resonance imaging [29].

This prospective observational study divided 100 patients with a first STEMI treated with pPCI divided into two groups: the remodeling group (*n* = 48) and the non-remodeling group (*n* = 52).

Diabetes, hypertension, dyslipidemia, and smoking did not differ significantly between the groups in this study. Arnautu et al. [30] performed case–control prospective research on 246 patients with LVEF ≥ 50% following PCI, dividing them into no LV remodeling group (*n* = 188) and LV remodeling group (*n* = 58). They reported that diabetes and heart rate differed insignificantly between the groups, but in their cohort, hypertension, smoking, SBP, and DBP did differ significantly between the groups. Chu et al. [31] also performed a prospective study; they divided 216 patients diagnosed with STEMI and treated with pPCI into non-remodeling group (*n* = 150) and remodeling group (*n* = 49). In their cohort, hypertension, dyslipidemia, and smoking differed insignificantly between groups, and diabetes was significantly higher in the remodeling group than non-remodeling group. Moreover, Shah et al. [32] enrolled 1,001 patients with a first acute STEMI (defined as ischemic chest pain ≥ 30 min and ST-elevation > 1 mm in ≥ 2 contiguous leads on the limbs or > 2 mm in ≥ 2 precordial contiguous leads) who were directed to a catheter lab for pPCI. They reported that diabetes, hypertension, dyslipidemia, and smoking all differed insignificantly between remodeling and non-remodeling groups. In the cohort of 85 patients with first acute anterior STEMI who underwent pPCI reported by Bastawy et al. [33], diabetes, hypertension, dyslipidemia, and smoking all differed insignificantly between the remodeling and non-remodeling groups. Those differences among studies can be explained by the different numbers of patients in each one.

In disagreement with our result, Eldamanhory et al. [34] performed a cross-sectional study of 82 patients who presented with a moderate risk of non-STEMI and were candidates for early invasive coronary angiography. All those patients were subjected to STE. In that cohort, diabetes, hypertension, and dyslipidemia were significantly higher in the non-remodeling group than the remodeling group. That difference can explained by the different patient conditions; in this study, we analyzed STEMI patients.

In this study, the door-to-balloon time was significantly lower in the non-remodeling group than the remodeling group. The initial LVESV and LVEDV were significantly higher in remodeling group than non-remodeling group, and the initial LVEF differed insignificantly between groups. The follow-up LVESV and LVEDV were significantly higher and LVEF was significantly lower in the remodeling group than the non-remodeling group. On the other hand, the percentage change in LVESV differed insignificantly between the groups, whereas the percentage change in LVEDV was significantly higher and the percentage change in LVEF was significantly lower in the remodeling group than the non-remodeling group. Therefore, patients in the remodeling group might have had more severe impairment of LV function than those in the non-remodeling group, which can result in increased LVESV and LVEDV. When the LV cannot pump blood efficiently, volumes can increase due to an accumulation of blood in the ventricle. In response to increased workload or injury, the LV can undergo compensatory changes, such as ventricular dilation, to maintain cardiac output. However, those compensatory mechanisms can eventually become maladaptive and contribute to further remodeling and increased LVESV and LVEDV [35].

Similarly, Arnautu et al. [30] reported that LVESV and LVEDV were significantly higher in the LV remodeling group than the non-remodeling group, and LVEF was significantly lower. Chu et al. [31] also reported that initial LVESV and LVEDV were significantly higher in the remodeling group than the non-remodeling group. However, in their cohort, initial LVEF was significantly higher in the non-remodeling group. Supporting our result, Shah et al. [32] reported that the percentage change in end-diastolic volume was significantly higher in the remodeling group than the non-remodeling group, whereas the percentage change in end-systolic volume differed insignificantly between the groups, and the percentage change in EF was significantly lower in the non-remodeling group. Those differences can be explained by the different number of patients in each study.

In our results, myocardial work differed significantly between the groups. GLS (initial, follow-up, and % change) was significantly higher in the remodeling group than the non-remodeling group. GWW (initial, follow-up, and % change) was significantly lower in the remodeling group than the non-remodeling group. GWI, GCW, and GWE (initial and follow-up) were significantly lower in the remodeling group than non-remodeling group, but their percentage changes were significantly higher in the remodeling group. In agreement with our results, Arnautu et al. [30] reported that GLS, GWI, GCW, GWW, and GWE all differed significantly between the non-remodeling and remodeling groups, and Chu et al. [31] also showed that GLS differed significantly between groups.

Hamada and Mansy [36] performed prospective cohort research with 136 participants with STEMI (defined as a 24-h emergence of chest discomfort). They reported that GLS was significantly higher in the remodeling group than the non-remodeling group. In our study, the initial LAr, LAcd, and LAct values differed significantly between the groups, The initial deceleration time, on the other hand, differed insignificantly between the groups. The initial LAV_maxBiP_ was significantly higher in the remodeling group, and the initial LAEF was significantly lower in the remodeling group than the non-remodeling group. The follow-up LAr, LAcd, and LAct values also differed significantly between the groups, and the follow-up LAV_maxBiP_ was significantly higher and follow-up LAEF was significantly lower in the remodeling group than the non-remodeling group. The percentage change in LAr, LAcd, and LAct differed significantly between the groups, and the percentage change of LAV_maxBiP_ was significantly higher and percentage change of LAEF was significantly lower in the remodeling group than the non-remodeling group.

In the remodeling group, LV dilatation and dysfunction can cause increased LA pressure and volume overload, leading to LA enlargement. An increase in LA volume is a marker of adverse cardiac remodeling and is associated with a high risk of adverse outcomes. B-type natriuretic peptide (BNP) is a hormone secreted by the heart in response to increased wall stress and volume overload. In the remodeling group, LV dilatation and dysfunction led to increased wall stress and volume overload, resulting in higher BNP levels. The follow-up measurements and percentage change in LAV_maxBiP_ were significantly higher in the remodeling group because cardiac remodeling increased LAV and BNP levels over time, compared with the non-remodeling group [37].

In our results, the mitral inflow peak early velocity/mitral annular peak early velocity was significantly higher in the remodeling group than the non-remodeling group. Moderate-to-severe mitral regurgitation did not occur in any patient. A higher E/e’ ratio suggests elevated LV end-diastolic pressure, which is a marker of diastolic dysfunction. After a STEMI, LV remodeling can lead to diastolic dysfunction, characterized by impaired LV relaxation and increased LV stiffness [27]. As a result, the mitral inflow E velocity increases due to higher filling pressures, and the mitral annular e’ velocity decreases due to impaired myocardial relaxation. A larger infarct size and more extensive myocardial ischemia in the remodeling group could contribute to more severe diastolic dysfunction and higher LV filling pressures, resulting in an elevated E/e’ ratio [38].

PALS, which is evaluated by speckle-tracking–derived strain, directly evaluates the atrial myocardium and offers a good reflection LA properties [39]. Bastawy et al. [33] showed that mitral E and mitral A differed insignificantly between remodeling and non-remodeling groups. Antoni et al. [40] confirmed the value of PALS for predicting adverse events in patients after an AMI was treated with PCI, and only 48 of 320 patients (15%) reached the composite endpoint. In this study, GLS showed promise as a predictor of LV remodeling in patients after STEMI, with 84.58% sensitivity and 64.23% specificity.

In agreement with our results, Chen et al. [41] reported that GLS was an independent predictor of LV remodeling, with an AUC of 0.775. Also supporting our results, Chu et al. [31] reported AUCs for LV GLS and PALS of 0.86 and 0.83, respectively. Interestingly, PALS did not add incremental value beyond LV GLS (AUC decreased from 0.86 to 0.83; *P* = 0.69) in predicting their composite event. The best cutoff values of LV GLS and PALS for LV remodeling in that study were –12.3% (sensitivity, 95.7%; specificity, 67.0%) and 28.9% (sensitivity, 88.1%; specificity, 65.2%), respectively. In the same line, Hamada and Mansy [36] showed that GLS can significantly predict early LV remodeling (AUC, 0.780; *P* < 0.001) at a cutoff of >–14, with 89.74% sensitivity, 77.32% specificity, 61.4% PPV, and 94.9% NPV, whereas LVEF could not predict early LV remodeling (AUC, 0.599; *P* = 0.055) at a cutoff of ≤ 50, with 46.15% sensitivity, 60.82% specificity, 32.1% PPV, and 73.7% NPV.

Bastawy et al. [33] reported that GLS differed significantly between groups, with higher values in the LV remodeling group. Their multivariate analysis showed that an average peak systolic GLS > − 12.5% was an independent predictor of LV remodeling (odds ratio, 1.7; 95% confidence interval, 0.4–13.8; *P* = 0.04). The best cutoff value for average peak systolic GLS was > − 12.5%, with 87% sensitivity, 85% specificity, 83% PPV, and 88% NPV. Also, Park et al. [42] demonstrated that GLS not only showed good predictive value for LV remodeling in patients with an anterior wall AMI, but also predicted death or heart failure as composite events, indicating that GLS is also a good predictor of adverse clinical events.

In this study, GCW emerged as the myocardial work parameter with the most significant association with LV remodeling post-STEMI, with 88.42% sensitivity and 77.38% specificity. GWW, GWE, and GWI also showed statistically significant associations with LV remodeling in patients after STEMI, with sensitivity of 64.58%, 60.42%, and 81.58%, respectively, and specificity of 38.46%, 36.54%, and 66.38%, respectively. In a previous study, greater reductions in GWI, GCW, and GWE and a greater increase in GWW were detected in STEMI patients who had developed ischemic LV remodeling at the 3-month follow-up visit [43]. Consistent with those results, El Mahdiui et al. [44] reported that GWE is lower in patients who have undergone pPCI intervention for STEMI than in healthy controls and those with cardiovascular risk factors and that GWE was even more impaired in the presence of heart failure with reduced EF. Those findings suggest that myocardial work impairment is the expression of altered (persistent anaerobic) energy metabolism in remodeled myocardium.

In the same line, Frișan et al. [45] reported that GCW showed the strongest association with adverse outcomes in the preserved LVEF group (AUC, 0.730; *P* = 0.035), and GWW demonstrated good discriminatory performance in the reduced LVEF group. That agreed with Chen et al. [41], who found that GWI was an independent predictor of LV remodeling, with an AUC of 0.806. Similarly, Arnautu et al. [30] reported that the most powerful predictor of LV remodeling was baseline GWE, with an AUC of 0.95, which was significantly higher than the AUC of the ROC curve for GWI (*P* < 0.04) and the AUC for the Killip class (*P* < 0.0001). They showed that the cutoff values predictive of LV remodeling were a baseline GWE ≤ 83% (sensitivity, 89.7%; specificity, 75.6%; *P* < 0.001), a baseline GWI ≤ 1,670 mmHg% (sensitivity, 82.7%; specificity, 76,4%; *P* < 0.0001), and a Killip class > 2 (sensitivity, 77.5%; specificity, 72.1%; *P* < 0.0001).

### Limitations

The double-center study design could limit the generalizability of our findings because patient populations and clinical practices vary across healthcare settings. Additionally, the relatively small sample size might have left our study underpowered to detect statistically significant differences between the patient groups.

The exclusion of non-STEMI patients restricts the comprehensiveness of the clinical profile assessed. Individuals with non-STEMI often exhibit characteristics distinct from those with STEMI, and incorporating a broader acute coronary syndrome population would enhance the applicability of the results.

Echocardiography was performed within 24 h post-pPCI. However, echocardiographic measurements obtained early after AMI can be influenced by transient myocardial stunning, dynamic loading conditions, acute ischemic injury, and hemodynamic variability.

## Conclusions

Myocardial work parameters (GLS, GWI, GCW, GWW, and GWE) were significantly associated with LV remodeling following STEMI treated with pPCI. Among those parameters, GCW demonstrated the strongest association with LV remodeling in the present cohort. LV remodeling was associated with adverse structural and functional cardiac changes, including higher LVESV and LVEDV, lower LVEF, and impaired myocardial work parameters. Additionally, LA function (LAr, LAcd, and LAEF) was significantly impaired in patients who developed LV remodeling.

## Data Availability

No datasets were generated or analysed during the current study.

## Abbreviations

AMI: Acute myocardial infarction
ASA: Acetylsalicylic acid
AUC: Area under the curve
BMI: Body mass index
BNP: B-type natriuretic peptide
DBP: Diastolic blood pressure
EF: Ejection fraction
GCW: Global constructive work
GLS: Global longitudinal strain
GWE: Global work efficiency
GWI: Global work index
GWW: Global wasted work
HbA1c: Hemoglobin A1c
LA: Left atrial
LAcd: Left atrial conduit function
LAct: Left atrial contraction
LAEF: Left atrial ejection fraction
LAr: Left atrial reservoir function
LAV_max_: Left atrial maximum volume at left ventricular end-systole
LAV_maxBiP_: Left atrial volume maximum biplane views
LAV_min_: Left atrial minimum volume at left ventricular end-systole
LAV_preA_: Left atrial volume before the active atrial contraction at the onset of the P wave
LPSS: Longitudinal peak systolic strain
LV: Left ventricular
LVEDV: Left ventricular end-diastolic volume
LVEF: Left ventricular ejection fraction
LVESV: Left ventricular end-systolic volume
NPV: Negative predictive value
PALS: Peak atrial longitudinal strain
pPCI: Primary percutaneous coronary intervention
PPV: Positive predictive value
ROC: Receiver operating characteristic
SBP: Systolic blood pressure
STE: Speckle-tracking echocardiography
STEMI: ST-elevation myocardial infarction
